# Professional Taxes

**Published:** 1878-09-20

**Authors:** 


### PROFESSIONAL TAXES.

There are in Georgia 1607 practicing physicians, of these 1255 are reported as paying the special tax of $10. There are 326 dentists, of whom only 179 are reported as having paid a professional tax. Thus it will be seen that while less than one-fourth of the physicians evade or fail to pay the speeific tax, that more than one-half of the destists escape it in some way.

The special tax upon physicians should be removed. At the late meeting of the Georgia Medical Association a committee was appointed to memoralize the Legislature for the repeal of the special tax on physicians.

A sufficient reason for making this request of the Legislature is that Medical men, *far more than all other callings combined*, contribute gratuitously their time, their medicines and their labor—not to mention their health and their comfort—to the necessities of the indigent sick; a burden which morally and legitimately attaches to the State authorities. The writer of this, once during an active village and country practice, kept an account of his gratuitous services and medicine to the poor for a period of twelve months, and it footed up the sum of $850 out of $1900 booked! We trust that physicians throughout the State will use their influence with their Representatives to secure the repeal of the professional tax on physicians.

